# ﻿*Ceratostema limonensis* (Ericaceae), a new species from the Province of Morona-Santiago, Ecuador

**DOI:** 10.3897/phytokeys.263.159645

**Published:** 2025-09-17

**Authors:** Marco M. Jiménez, Gabriel A. Iturralde, Diego Gutiérrez del Pozo, Nadia Lapo-González, J. R. Kuethe, Henry X. Garzón-Suárez

**Affiliations:** 1 Grupo de Investigación en Biodiversidad, Medio Ambiente y Salud BIOMAS, Carrera de Ingeniería Agroindustrial, Facultad de Ingeniería y Ciencias Aplicadas, Universidad de Las Américas, UDLA, Vía a Nayón, Quito 170124, Ecuador Universidad de las Américas Quito Ecuador; 2 Universidad Estatal Amazónica UEA, Herbario Amazónico del Ecuador ECUAMZ. Carretera Tena a Puyo Km. 44, Napo, Carlos Julio Arosemena Tola 150950, Ecuador Universidad Estatal Amazónica Carlos Julio Arosemena Tola Ecuador; 3 Grupo Científico Calaway Dodson: Investigación y Conservación de Orquídeas del Ecuador, Quito, 170510, Pichincha, Ecuador Grupo Científico Calaway Dodson: Investigación y Conservación de Orquídeas del Ecuador Quito Ecuador; 4 Floplaya, Compañía de flores y plantas Yantzaza S.A., El Pangui, 190650, Ecuador Floplaya, Compañía de flores y plantas Yantzaza S.A. El Pangui Ecuador; 5 School of Environment, Faculty of Sciences, University of Auckland, City Campus for Science and Engineering, Symonds Street, Auckland, New Zealand University of Auckland Auckland New Zealand; 6 Herbario HUTPL, Departamento de Ciencias Biológicas, Universidad Técnica Particular de Loja, San Cayetano Alto s/n 11-01-608, Loja, Ecuador Universidad Técnica Particular de Loja Loja Ecuador; 7 Jungle Dave’s Science Foundation, San Juan Bosco, Ecuador Jungle Dave’s Science Foundation San Juan Bosco Ecuador

**Keywords:** Amazon, Andes, premontane forest, rainforest, taxonomy

## Abstract

A new species of *Ceratostema* (Vaccinieae) from south-eastern Ecuador is here described and illustrated. *Ceratostema limonensis* is distinguished by the singular, subflexuous young branches. The new species is compared to the morphologically similar *C. gualaquizensis*, which differs by the subterete, longer rachis and shorter corollas with narrower monochromatic corolla lobes. A brief history of the species from the tribe Vaccinieae in Ecuador is provided. Further taxonomic discussions, distribution and conservation status for the new species are provided in this paper.

## ﻿Introduction

Members of the heather family (Ericaceae Juss.) can be found on all continents (except Antarctica), where it reaches its greatest diversity within the tropical latitudes, such as the mountains of the tropical Andes. Currently, this family is divided into eight subfamilies (Kron et al. 2002), with Vaccinieae being the most represented across Ecuador. This group is foremost characterised by having inferior ovaries and edible fruits and, for this reason, are of local economic importance, being cultivated as small fruit crops, but also in ornamental value (Luteyn 2021). Seventeen genera of Vaccinieae are currently known for Ecuador, with the largest number of species present within the genus *Ceratostema* Juss. (Luteyn 2021). Initially encompassing 28 species (Luteyn 1996), targeted studies conducted during recent years have increased this number to more than 40, making Ecuador amongst the most biodiverse countries for this genus (Jiménez et al. 2021; Cornejo and Luteyn 2024; Cornejo et al. 2024; Doucette et al. 2024; Jiménez et al. 2024a, 2024b, 2024c; Doucette et al. 2025a, 2025b, 2025c).

*Ceratostema* was originally established by de Jussieu (1789) and a few attempts have been made since the 20^th^ century to divide the genus into independent genera, namely *Englerodoxa* Hoerold and *Periclesia* A.C.Sm. (Hoerold 1909; Smith 1942). Previous writings, including Smith (1952), recognised 16 species of *Ceratostema* during the mid-1900s; the number was increased to 23 by Luteyn (1986) three decades later. In 1996, Luteyn published a national survey focusing on the *Ceratostema* present in Ecuador, including a key for the species and introducing nine new species present within this country. Subsequently, Pedraza-Peñalosa et al. (2015) used molecular studies to suggest that *Ceratostema* is a polyphyletic group, but did not propose an infrageneric classification to better establish an intrageneric understanding of *Ceratostema* as a whole.

*Ceratostema* are typically represented by terrestrial plants or epiphytes that frequently arise from lignotubers with alternate, petiolate leaves. Flowers are sympetalous, bearing a lower ovary and stamens that are usually as long as the corolla. The long anther tubules and calyx are articulated with the pedicel and the basal corolla is divided into lobes that are proportionally more elongated than the rest of its length (Luteyn 1986; Luteyn 1996; Cornejo and Luteyn 2024).

Recent fieldwork carried out by the authorial team in the south-eastern Andes of Ecuador has uncovered yet another new species of *Ceratostema* for Ecuador. This new species was found during botanical studies carried out in the Morona-Santiago Province. It was seen growing in the evergreen forests of the superhumid regions of the eastern slope of the Andes, a region classed as one of the 25 biodiversity hotspots exhibiting some of the richest biotas of flora and fauna on Earth (Myers et al. 2000; Killeen et al. 2007). Unfortunately, a high degree of deforestation and land degradation poses questions about the long-term survival of this species and conservation efforts should be undertaken to prevent further decline of these important habitats. Notes regarding the distribution, natural habitat and conservation status of this new species have been provided in this paper.

## ﻿Materials and methods

This new species of *Ceratostema* was discovered by the authors (MJ and HG) during botanical field explorations conducted between 2021 and 2025 in the south of the Morona-Santiago Province. The species was encountered during ongoing floristic surveys focused on poorly-known montane ecosystems present within the region.

Taxonomic evaluation was conducted through detailed comparisons with the known described species of *Ceratostema* from eastern Ecuador, using specialised literature for the Ecuadorian taxa (e.g. Luteyn (1996); Cornejo and Luteyn (2024); Cornejo et al. (2024); Jiménez et al. (2024b); Doucette et al. (2025c)) and a comparative study utilising online herbarium databases including Tropicos, JSTOR Global Plants and SpeciesLink. Morphological terminology follows Beentje (2016).

Photographs were taken in situ using a Nikon® D3100 camera, equipped with an AF-S VR Micro-Nikkor 40 mm f/2.8G IF-ED lens and a Raynox® DCR-250 mm Super Macro lens, supported by two Nikon® SB-700 AF speed-light flashes. Herbarium specimens were collected, pressed and deposited at the Herbarium of the Universidad Técnica Particular de Loja (HUTPL). Floral structures were dissected manually and macro-photographed in the field, including metric scale references and then preserved in 70% ethanol with 1% glycerine. ImageJ software was applied for obtaining measurements from fresh specimens and high-resolution photographs (https://ij.imjoy.io/). Illustrations were prepared using Adobe® Photoshop CS6. A distribution map was generated in ArcGIS Desktop 10.3.

All fieldwork and specimen collection were conducted under permit No. MAATE-DBI-CM-2022-0248, issued by the Ecuadorian Ministry of Environment and Ecological Transition (MAATE).

## ﻿Taxonomic treatment

### ﻿ Ceratostema limonensis

Taxon classificationPlantaeEricalesEricaceae

M.M.Jiménez & H.Garzón sp. nov.

A2628450-5379-5E5F-AF1D-340F78C7D82E

urn:lsid:ipni.org:names:77369307-1

Figs 1, 2, 4

#### Diagnosis.

*Ceratostema limonensis* is morphologically similar to *C. gualaquizensis*, but differs by the longer (4.8–5.1 mm vs. 1 mm long), subterete (vs. obconical) rhachis; the shorter (1.4–1.8 mm vs. 3.4–4.7 mm long), ovate-deltate (vs. narrowly lanceolate) calyx lobes; the shorter corolla (2.9 cm vs. 4.5–4.7 cm long) with narrower lobes (2.3 mm vs. 3.7–4.0 mm wide), that have the same colour as the rest of the corolla (vs. black at the apex and inside); and the shorter (3.1–3.3 cm vs. 4.6–4.7 cm long), pilose (vs. glabrous) filaments.

#### Type.

Ecuador • Morona-Santiago: Cerca de Limón, 1298 m alt., 28 April 2025, *H. Garzón 293* (holotype: HUTPL!).

#### Description.

Pendant, epiphytic ***shrubs***; indumentum consisting of short, white, almost persistent, eglandular trichomes of 0.2–0.7 mm long, trichomes arranged unevenly, sparsely to densely on younger branches, petioles, leaf blades, inflorescences and flowers, including stamens and style; axonomorphous roots with well-developed lignotubers, lignotubers fusiform, 6.0–7.3 × 2.1–2.8 cm. ***Stems*** terete to subterete, 5–8 cm long, glabrous, slightly arching, arising from the lignotuber, the older stems dark brown, cracking longitudinally and exfoliating; younger branches subterete, up to 18 cm long, 0.8–1.3 mm wide, pendant, subflexuous, dark brown, pilose, becoming glabrous and striate when old or after exfoliation; axillary buds emerging 1 mm below the leaf node. ***Leaves*** spirally arranged, subsessile, pendulous to almost horizontal; petioles subterete, 0.9–1.9 × 1.0 mm, pilose, pale green; blades lanceolate, 5.5–7.8 × 1.8–2.0 cm, thinly-coriaceous, convex with the basal margins folded to conceal flowers and fruits, base cordate to obtuse, apex acuminate, dark green adaxially, pale green abaxially, lustrous and pilose adaxially, dull and pilose abaxially, becoming glabrous with age, 5–7-plinerved from near the base, the mid-vein raised along almost its length adaxially, thickened in the proximal 12 mm, impressed and hollow abaxially, the secondary veins raised adaxially, weakly impressed abaxially, branching, anastomosing distally with reticulate veinlets, slightly raised adiaxally, obscure abaxially. ***Inflorescence*** axillary, 1–2-flowered, sessile; rachis subterete, constricted in the middle, 4.8–5.1 mm long, 1.4 mm thick, subverrucose, covered at the base by several bracts; bracts deltate, 0.4–0.6 mm long, minute, persistent, whitish-green, pilose, acute; floral bracts narrowly ovate, ca. 0.6 × 0.2 mm, suffused with pink, pilose, attenuate; pedicel subclavate, 5.1–5.5 mm long, 2.6–2.8 mm thick, subverrucose, slightly arcuate, pale green, pilose, articulate with the calyx; bracteoles 2, ovate-triangular, 0.5–0.7 × 0.3–0.5 mm, minute, whitish-green, pilose, attenuate, located near the base and opposite. ***Flowers*** pentamerous rarely tetramerous, descending to pendulous; calyx pilose, 5.1–6.0 × 4.5–5.0 mm, green; hypanthium obconic, 2.7–3.8 × 4.6–5.0 mm, obscurely 5-ridged, truncate; limb open, 1.9 × 4.2–4.5 mm, erect; lobes 5, ovate-deltate, 1.4–1.8 × 1.8–2.1 mm, small, shortly acuminate, the sinuses acute. ***Corolla*** tubular, slightly narrowing distally, 2.9 cm long (including the lobes), thick-carnose, bistratose, pubescent and bluntly 5-angled along its length, pubescent in the internal apical half, 5 mm in diameter at the base, 4 mm in diameter at the throat, magenta, paler at the angles; lobes 5, narrowly linear-triangular, 14.0–15.7 × 2.3 mm, spreading, acuminate, slightly incurved, glabrous, channelled and subverrucose internally. ***Stamens*** 10, nearly equalling the corolla in overall length, each pair unequal, 3.1–3.3 cm long; filaments connate forming a tubular staminal tube, slightly dilated distally, 8.9–9.6 mm long, white with a touch of pink, pilose externally in the proximal half; anthers 2.3–2.7 cm long, thecae conspicuously granulose, 6.2–6.8 mm long, prognathous, each pair of thecae unequal, 4.8–5.0 mm long; tubules distinct, 1.8–2.2 cm long, slightly unequal, seemingly connate in almost its length, straight, glabrous, dehiscing by terminal pores, 0.6 × 0.3 mm. ***Style*** exserted, 2.9–3.4 cm long, sparsely pilose at the middle, pale green, stigma truncated. ***Fruits*** not seen.

**Figure 1.**
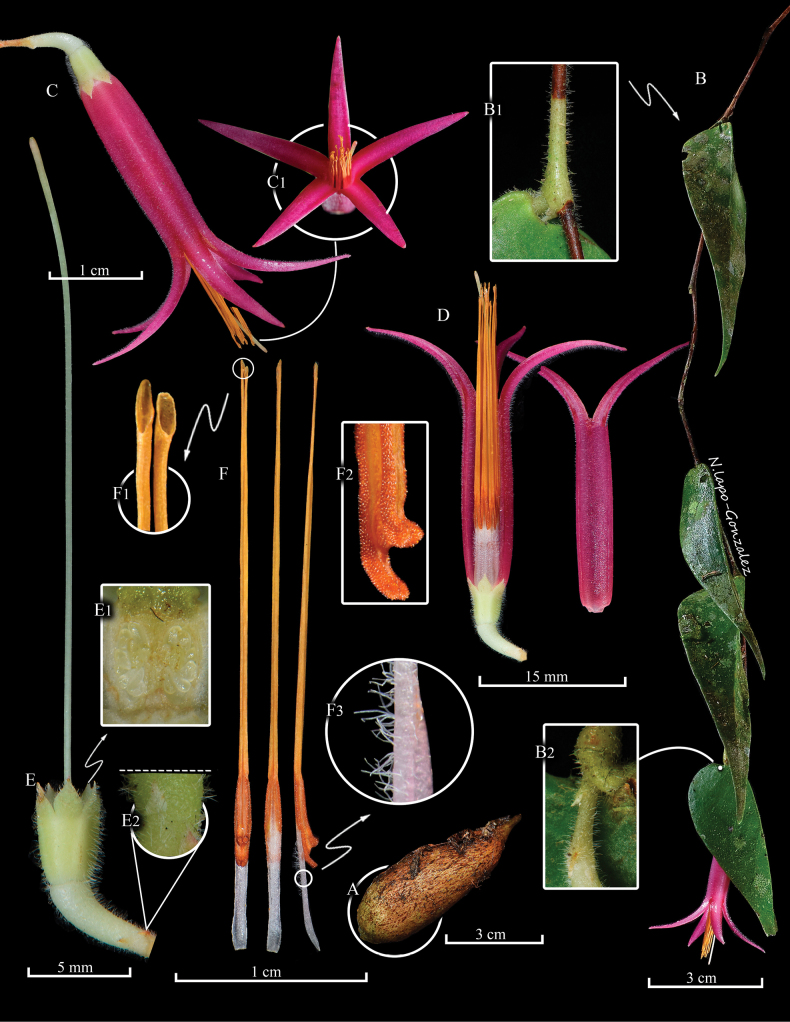
Lankester Composite Dissection Plate (LCDP) of *Ceratostema limonensis*. A. Lignotuber; B. Fertile branch with a close-up of the node (B1) and rachis of the inflorescence (B2); C. Complete flower with a ventral view of the corolla lobes (C1); D. Flower with a longitudinal section of the corolla showing stamens and the corolla without stamens (right); E. Calyx, pedicel and style with a close-up of the ovary (E1) and the bracteole (E2); F. Stamens with a close-up of the pores of the tubule (F1), anther thecae (F2) and external surface of the filament (F3). Prepared by N. Lapo-González from photographs of the holotype.

**Figure 2.**
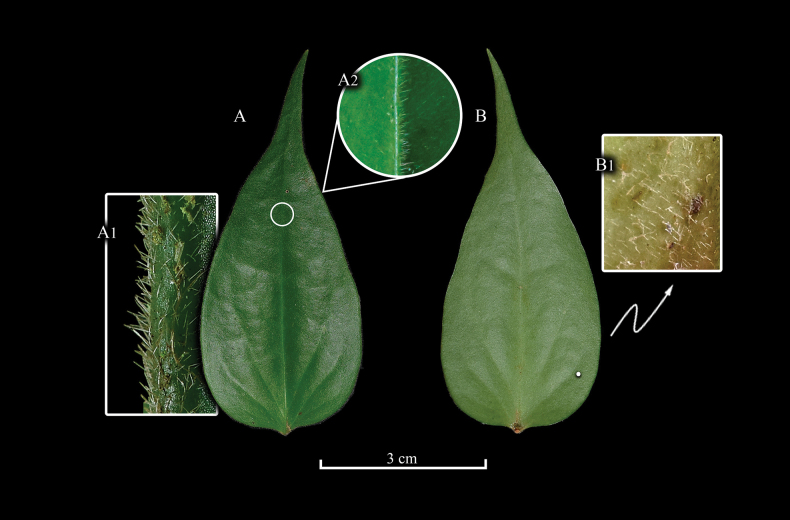
Leaves of *Ceratostema limonensis* with close-ups of the indumentum. A. Adaxial view with a close-up of the margin (A1) and mid-vein (A2); B. Abaxial view with a close-up of the basal portion of the leaf (B1). Prepared by N. Lapo-González from photos by H.X. Garzón-Suárez.

#### Distribution and habitat.

**Figure 3.**
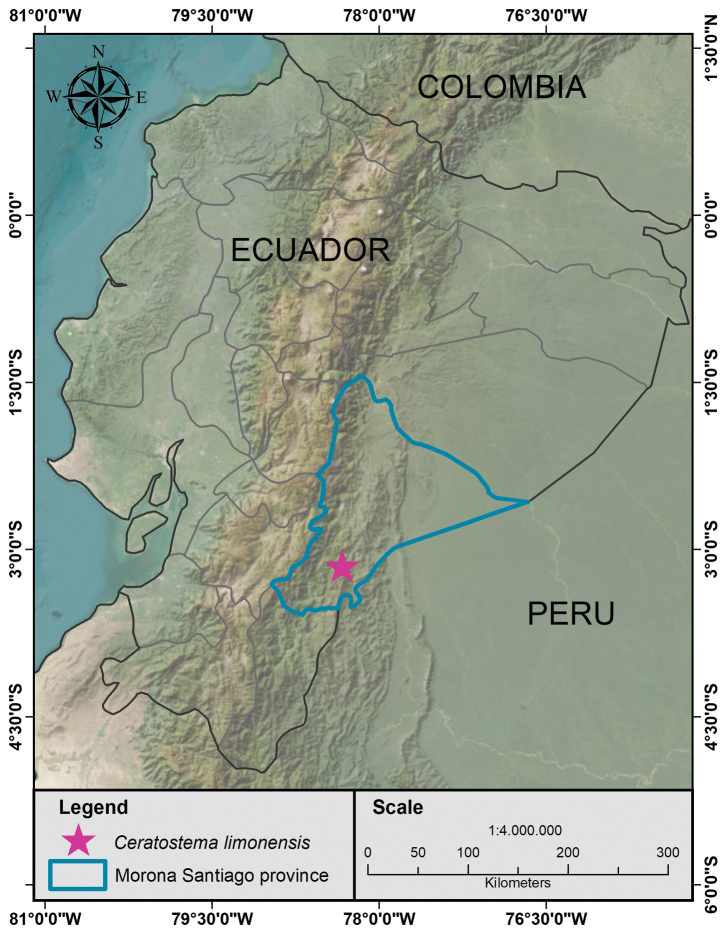
Distribution of *Ceratostema limonensis* in the south-eastern region of Ecuador.

**Figure 4.**
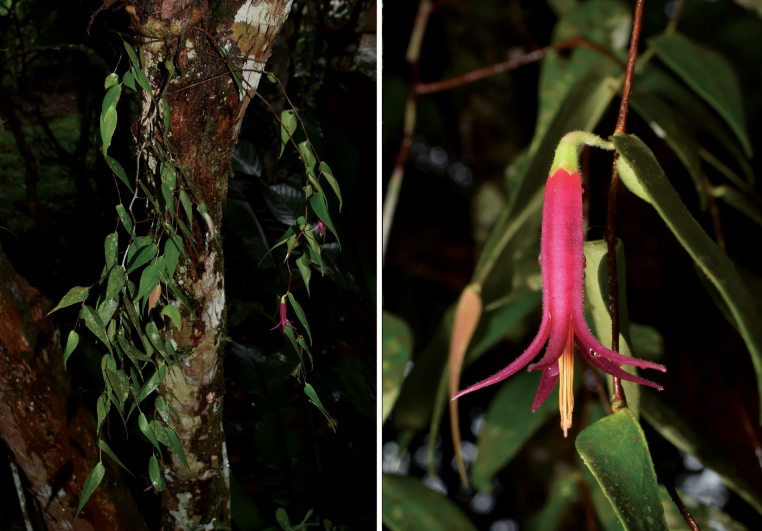
*Ceratostema limonensis* in situ. Photos by Henry X. Garzón-Suárez.

*Ceratostema limonensis* is currently known only from the vicinity of Limón in southern Morona-Santiago Province, where it was found on the south-eastern slopes of the Ecuadorian Andes (Fig. 3). It has been observed at elevations between 1100 and 1400 m in habitats characterised by mature, lower montane forests (Fig. 4). These forests are part of the “Bosque siempreverde piemontano del sur de la Cordillera Oriental de los Andes” (code BsPn04), as defined by the Ecuadorian Ministry for Environment (Ministerio del Ambiente del Ecuador 2013). The forest at the type locality is multi-stratified with a semi-open forest canopy and few lower forest glades with a maximum height of ca. 20 m. The most dominant species are *Alchornea grandis* Benth., Ficus cf. trapezicola Dugand, *Metteniusa tessmanniana* (Sleumer) Sleumer and *Otoba parvifolia* (Markgr.) A.H.Gentry. Specimens of *C. limonensis* were found sympatric with other flora, such as *Evodianthus funifer* Lindm., *Maxillaria mapiriensis* (Kraenzl.) L.O.Williams, *Ronnbergia campanulata* Gilmartin & H.Luther and *Stenospermation* sp.

#### Etymology.

This species is named after the Municipality of Limón in the Province of Morona-Santiago, the township near to which the new species was discovered.

#### Conservation status.

The very limited distribution of *Ceratostema limonensis* suggests that this species is a narrow endemic to south-eastern Ecuador. Its low abundance, combined with its restricted occurrence, does not allow for assessment of the AOO or EOO at this stage. Conservation threats to this species are excessive farming and mining activities which already occurring near the type locality. Due to the absence of more known specimens belonging to this species, a tentative classification of Data Deficient (DD) has been given (IUCN 2024).

#### Taxonomic discussion.

*Ceratostema limonensis* is primarily distinguished from all other species within the genus *Ceratostema* by its subflexuous young branches and axillary inflorescences, characterised by an entire, subterete rachis and pedicel. The inflorescences likely produce a second successive flower, a trait observed in related species, such as *C. alexportillae* A.Doucette, H.Medina & J.Portilla, *C. gualaquizensis* M.M.Jiménez & H.Garzón, *C. loucianae* Cornejo, G.Tello & Luteyn and *C. rauhii* Luteyn; however, further data on the floral production of this new species are necessary. These species also share several morphological features, including an epiphytic, pendant growth habit; spirally arranged, subsessile, plinerved, acuminate leaves; subsessile inflorescences with minute floral bracts and bracteoles; dark-coloured corollas with lobes that are narrowly triangular to narrowly linear-triangular, acuminate and recurved; and an exserted style (Luteyn 1992; Cornejo and Luteyn 2024; Cornejo et al. 2024; Jiménez et al. 2024b; Doucette et al. 2025a).

Amongst these, *Ceratostema limonensis* most closely resembles *C. gualaquizensis*, as both possess non-amplexicaul, plinerved leaves folded at the base to enclose magenta flowers. Their hypanthia are similarly obconic and subtly 5-ridged, adorned with acuminate calyx lobes. However, they differ in the characteristics set out in the diagnosis and, in addition, in that *C. limonensis* has foliar veins raised adaxially, whereas in *C. gualaquizensis*, the veins are impressed in the proximal 4 mm. Additionally, the corolla lobes of *C. limonensis* comprise approximately 50% of the total corolla length, compared to about 30% in *C. gualaquizensis*. *C. limonensis* also shares similarities with *C. alexportillae*, lianoid branches and notably convex, lanceolate leaves folded at the base (Jiménez et al. 2024b; Doucette et al. 2025a). A comparative summary of morphological differences amongst these species is provided in Table 1.

**Table 1.** Morphological comparison of *Ceratostema limonensis* and similar species. References taken from: (1) presented herein, (2) Jiménez et al. (2024b) and Cornejo and Luteyn (2024); (3) Doucette et al. (2025a); (4) Jiménez et al. (2024b); (5) Luteyn (1992).

Characters	C. limonensis (1)	C. gualaquizensis (2)	C. alexportillae (3)	C. loucianae (4)	C. rauhii (5)
Indumentum on vegetative and floral parts	Pilose, pubescent on the corolla	Puberulous	Glabrous	Villose	Puberulous
Branches	Lianoid	Filiform	Lianoid	Long-filiform	Long-filiform
Leaf blades					
Shape	Lanceolate, convex with basal margins folded	Elliptic, convex with basal margins folded	Cordate, convex with basal margins folded	Ovate, flat-canaliculate	Narrowly ovate to linear-ovate, flat
Length (cm)	5.5–7.8	3.9–6.0	4.8–7.2	2.5–6.0	1.5–3.5
Width (cm)	1.8–2.0	2.2–2.5	Ca. 0.2	1.5–3.3	0.5–0.7
Venation	5–7-plinerved	5–7-plinerved	Obscure	5–7-plinerved	3–5-plinerved
Base	Cordate to obtuse, non-amplexicaul	Obtuse, non-amplexicaul	Cordate, amplexicaul	Subcordate to truncate, non-amplexicaul	Obtuse, non-amplexicaul
Rachis					
Shape	Subterete	Obconical	Obconical	Obconical	Not available
Length (mm)	4.8–5.1	Ca. 1.0	Ca. 3.8	Ca. 1.0	Not available
Pedicel					
Shape	Subclavate	Subterete	Terete-conical	Subterete	Terete
Length (mm)	5.1–5.5	5.0–6.0	4.9–5.0	6.0–11.0	Ca. 2.0–3.0
Hypanthium					
Shape	Obconical, obscurely 5-ridged	Obconical, obscurely 5-ridged	Broad obconical, 5-carinate	Obconical, subterete to bluntly 5-costate	Cylindrical to obconical, terete
Length (mm)	2.7–3.8	3.9–4.2	3.2–3.7	2.9–3.5	Ca. 2.0
Calyx lobes					
Shape	Ovate–deltate	Narrowly lanceolate	Broadly deltoid	Broadly deltoid	Narrowly triangular
Length (mm)	1.4–1.8	3.4–4.7	Ca. 1.0	0.7–0.8	3.5–4.5
Apex	Shortly-acuminate	Acuminate	Apiculate	Apiculate	Long-acuminate
Sinuses	Acute	Acute	Obtuse	Rounded	Acute
Corolla					
Colour	Magenta, paler at the angles	Magenta	Red-purple	Red and black	Bright red
Shape	Tubular, bluntly 5-angled	Tubular, obscurely to bluntly 5-angled	Tubular, pentagonal	Cylindrical-urceolate to tubular, bluntly 5-angled	Tubular, bluntly 5-angled
Length (mm)	Ca. 29.0	45.0–47.0	Ca. 29.0	18.0–28.0	28.0–30.0
Corolla lobes					
Colour	Magenta	Magenta and black	Purple-black	Black	Bright red
Shape	Narrowly linear-triangular	Narrowly triangular	Narrowly triangular	Narrowly linear-triangular	Narrowly triangular
Length (mm)	14.0–15.7	12.1–17.0	Ca. 12.5	11.5–17.8	3.5–4.5
Apex	Acuminate	Acuminate	Acute	Acuminate	Long-acuminate
Stamens					
Length (cm)	3.1–3.3	4.4–4.6	2.3–2.4	2.6–3.1	Ca. 2.8
Filaments texture	Pilose	Glabrous	Sparsely pubescent	Glabrous	Glabrous
Thecae length (mm)	6.2–6.8	Ca. 5.1 mm	3.8–4.2	6.0–10.0	Ca. 4.0
Tubules shape	Straight	Straight	Sinuous	Straight	Straight
Tubules length (cm)	1.8–2.2	3.4–3.5	Ca. 1.6	1.2–1.5	Ca. 1.8
Style					
Length (cm)	2.9–3.4	4.8–5.1	Ca. 3.0	3.3–3.4	2.8–3.0
Texture	Sparsely pilose	Glabrous	Glabrous	Glabrous	Glabrous

## Supplementary Material

XML Treatment for Ceratostema limonensis

## ﻿Additional information

### Use of AI

No use of AI was reported.

### Author ORCIDs

Marco M. Jiménez https://orcid.org/0000-0002-9502-5651

Gabriel A. Iturralde https://orcid.org/0000-0003-2456-0929

Diego Gutiérrez del Pozo https://orcid.org/0000-0003-3611-7932

Nadia Lapo-González https://orcid.org/0009-0001-8863-1557

J. R. Kuethe https://orcid.org/0000-0001-5390-9654

Henry X. Garzón-Suárez https://orcid.org/0000-0003-4049-1652

### Data availability

All of the data that support the findings of this study are available in the main text.
